# Kinetics of IL-6 Production Defines T Effector Cell Responsiveness to Regulatory T Cells in Multiple Sclerosis

**DOI:** 10.1371/journal.pone.0077634

**Published:** 2013-10-14

**Authors:** Bettina Trinschek, Felix Lüssi, Jürgen Haas, Brigitte Wildemann, Frauke Zipp, Heinz Wiendl, Christian Becker, Helmut Jonuleit

**Affiliations:** 1 Department of Dermatology, University Medical Center of the Johannes Gutenberg-University Mainz, Mainz, Germany; 2 Department of Neurology, University Medical Center of the Johannes Gutenberg-University Mainz, Mainz, Germany; 3 Division of Molecular Neuroimmunology, Department of Neurology, University of Heidelberg, Heidelberg, Germany; 4 Department of Neurology-Inflammatory Disorders of the Nervous System and Neurooncology, University of Muenster, Muenster, Germany; Chinese Academy of Sciences, China

## Abstract

In multiple sclerosis (MS) autoaggressive T effector cells (Teff) are not efficiently controlled by regulatory T cells (Treg) but the underlying mechanisms are incompletely understood. Proinflammatory cytokines are key factors facilitating Teff activity in chronic inflammation. Here we investigated the influence of IL-6 on Treg sensitivity of Teff from therapy-naïve MS patients with or without active disease. Compared to healthy volunteers and independent of disease course CD4^+^ and especially CD8^+^ MS-Teff were insensitive against functional active Treg from healthy controls. This unresponsiveness was caused by accelerated production of IL-6, elevated IL-6 receptor expression and phosphorylation of protein kinase B (PKB)/c-Akt in MS-Teff. In a positive feedback loop, IL-6 itself induced its accelerated synthesis and enhanced phosphorylation of PKB/c-Akt that finally mediated Treg resistance. Furthermore, accelerated IL-6 release especially by CD8^+^ Teff prevented control of surrounding Teff, described here as “bystander resistance”. Blockade of IL-6 receptor signaling or direct inhibition of PKB/c-Akt phosphorylation restored Treg responsiveness of Teff and prevented bystander resistance. In Teff of healthy controls (HC) exogenous IL-6 also changed the kinetics of IL-6 production and induced Treg unresponsiveness. This modulation was only transient in Teff from healthy volunteers, whereas accelerated IL-6 production in MS-Teff maintained also in absence of IL-6. Hence, we showed that the kinetics of IL-6 production instead of elevated IL-6 levels defines the Teff responsiveness in early Treg-T cell communication in MS independent of their disease course and propose IL-6 and associated PKB/c-Akt activation as effective therapeutic targets for modulation of Teff activity in MS.

## Introduction

T effector cell (Teff) control by Foxp3^+^ regulatory T cells (Treg) in the periphery is crucial for the maintenance of immune homeostasis. This peripheral tolerance is directly or indirectly evoked through several ways. Thymus-derived Foxp3^+^ Treg conduct their suppressive function via direct cell contact [1]. In contrast, periphery-derived Treg mediate suppressive effects also by production of cytokines like TGF-β or IL-10 that allow cell contact-independent suppression and transfer of suppressive properties to other T cells, a process termed infectious tolerance [2,3]. This homeostasis which is maintained by mechanisms of peripheral tolerance can be biased by the influence of pro- and anti-inflammatory cytokines.

A prototypic proinflammatory cytokine associated with the pathology of several diseases is IL-6. It has a key function in immune responses, inflammation, hematopoiesis and acute phase responses [4]. Dysregulated IL-6 is connected with the pathogenesis of various chronic autoimmune disorders including rheumatoid arthritis (RA), Crohn’s disease and type 1 diabetes, but also cancer [5-8]. T cells are both, main source and important target of IL-6. Together with TGF-β, IL-6 promotes Th17 differentiation [9-12] and inhibits generation of induced Treg [13].

Therefore, modulation of IL-6 or downstream signals has become a promising strategy to control autoimmune diseases [14]. Blockade of IL-6 in rheumatoid arthritis patients led to reduced disease activity and substantial improvement in clinical signs further strengthening the therapeutic potential of IL-6 modulation [15]. Finally, this resulted in the approval of Tocilizumab, an IL-6-blocking antibody for RA treatment.

In a similar way as in RA, IL-6 also influences the development and onset of experimental autoimmune encephalomyelitis, the murine model for multiple sclerosis (MS) [16,17]. Although IL-6 levels in MS patients could not be associated with disease activity [18], its production by astrocytes in the CNS at the site of demyelination and in acute and chronic active lesions [19] suggests a participation of IL-6 in MS pathogenesis [18,19]. More recently it was shown that Teff from relapsing remitting MS patients (RRMS) with active disease are not efficiently controlled by Treg. This unresponsiveness in some cases correlated with enhanced IL-6 levels [20]. Since these patients had an active disease they were exposed to a variety of cytokines and chemokines that maintain the inflammatory process and influence Teff responsiveness to Treg. Up to now Teff resistance and enhanced IL-6 levels were only observed in MS patients with active disease or with relapses [20], but not in patients in remission. Collectively, these results increase the evidence that IL-6 plays a central role in the pathogenesis of T cell-mediated autoimmunity, but the underlying mechanisms remain incompletely understood.

Here, we studied the influence of IL-6 on T cell immune regulation in RRMS patients in remission and observed a new mechanism in which the pleiotropic cytokine IL-6 when present at early stages of T cell activation induces a positive feedback loop finally leading to unresponsiveness against Treg-mediated control. In agreement with others we did not observe a significant enhancement of IL-6 synthesis but we found an accelerated IL-6 kinetics in activated Teff from therapy-naïve MS patients without active disease. These Teff were insensitive to Treg-mediated suppression which essentially depend on their accelerated kinetics of IL-6 synthesis and constitutive IL-6R expression. Early IL-6 synthesis especially by activated CD8^+^ Teff from MS patients also conveyed Treg insensitivity to surrounding T cells, a process we described as “bystander resistance”. Furthermore, IL-6 itself accelerated its production in CD4^+^ and CD8^+^ T cells. Thus, we conclude that IL-6 triggers a positive feedback loop enhancing IL-6 production by T cells and conferring a state of insensitivity to Treg function.

## Methods

### Patients and healthy controls

This study was approved by the local ethical committee (Landesaerztekammer Rheinland-Pfalz). 51 patients with a relapsing-remitting course (RRMS, age 21 to 64 years) fulfilling the revised McDonald criteria for multiple sclerosis [21] and were included in this study. All patients had not received previous treatment or immunosuppressive agents six months before time point of analysis and were clinically stable. PBMC from 72 healthy individuals served as controls. According to the principles expressed in the Helsinki Declaration and to approved protocols patients provided written informed consent before participating in this study. 

### Culture Medium and Antibodies

Human cells were cultured in X-VIVO-15 (Lonza). Flow cytometric analysis was performed using the following antibodies. Anti-human CD3 (SK7), anti-human CD3 (UCHT1), anti-human CD4 (RPA-T4), anti-human CD8 (SK1), anti-human CD14 (M5E2), anti-human CD19 (HIB19), anti-human CD25 (M-A251), anti-human IL-6 (MQ2-13A5), anti-(pS473) pPKB/c-Akt (M89-61), all from BD Pharmingen, anti-GARP (G14D9, eBioscience), anti-CTLA-4 (BNI3, BD eBioscience), anti-human CD8 (BW 135/80, Miltenyi Biotec), Fluorokine® biotinylated human Interleukin-6 (R&D systems). Cell viability during flow cytometric analysis was determined using 7-AAD (eBioscience). For blockade experiments, cultures were supplemented with neutralizing antibody against anti-IL-6R (Tocilizumab) or PKB/c-Akt VIII inhibitor (Calbiochem).

### Flow cytometry

For surface staining of PBMC or T cells indicated antibodies were incubated for 30 min. at 4 °C and washed twice with PBS. Stained cells were measured on LSRII with FACS Diva Software (BD Bioscience). To detect phosphorylated PKB/c-Akt, cells were fixed at 37 °C (BD Cytofix^TM^ Buffer); permeabilized (BD^TM^ Phosflow Perm Buffer) washed twice with BD Pharmingen^TM^ Stain buffer and stained for the indicated antibody according to manufacturer`s instructions.

### Isolation of T cell subsets

CD4^+^CD25^+^Foxp3^+^ Treg were isolated from PBMC using anti-CD25 MicroBeads (Miltenyi Biotec) and depleted of contaminating CD8^+^, CD14^+^ and CD19^+^ cells with Dynabeads (Invitrogen) as described previously [22]. Purity was routinely >80 %, Treg functionality was ensured in standard suppressor assays. Untouched CD3^+^ T cell isolation was performed using pan T cell isolation kit (Miltenyi Biotec) according to manufacturer’s instructions. For some experiments PBMC were depleted of CD3, CD19 or CD25 using corresponding Dynabeads (1 bead/cell; Invitrogen).

### Cytokine analysis

Treg-depleted PBMC from HC or MS patients were cultured in presence or absence of Treg (ratio 1:1) and stimulated with anti-CD3 mAb (OKT3). Cytokines in supernatants were measured 72 h after stimulation by Cytometric Bead Array (BD Bioscience) following manufacturer’s instructions and analyzed by GraphPad Prism6 (Statcon). For intracellular cytokine staining anti-IL-6-APC was used. PBMC of either HC or MS were activated with 1 µg/ml Ionomycin and 1 ng/ml PMA for 5 h, 4 h in the presence of Monensin (1.3 µM/ml). After stimulation cells were collected, washed, permeabilized (perm/fix solution; BD Pharmingen) and stained for above mentioned cytokine.

### Suppressor assays

Treg-depleted PBMC (10^5^ cells) were stimulated with 0.5 µg/ml anti-CD3 mAb (OKT3) and cultured in presence or absence of different Treg ratios (Treg:Teff 1:1 to 1:64) [22,23]. Teff proliferation was determined on day three of cultures by addition of 37 kBq/well ^3^H-Tdr for additional 16 h. Some experiments were performed by supplementing cultures with neutralizing mAb against anti-IL-6R (30 ng/ml; Tocilizumab; Roacterma; Roche) or supplemented with IL-6 (100 ng/ml or 1000 IU/ml; CellGenix). PKB/c-Akt VIII inhibitor (0.1 µM; Calbiochem) was added into individual assays. Untouched CD3^+^ Teff were isolated by pan T cell isolation kit (Miltenyi Biotec) and stimulated with CD3-depleted and irradiated PBMC of independent 3^rd^ donors and 0.5 µg/ml anti-CD3 mAb in presence or absence of different Treg ratios (Treg:PBMC 1:1-1:64). For flow cytometric analysis of proliferating T cells, Treg-depleted PBMC were washed in warm PBS and stained with 1 µM CFSE and afterwards cocultured with or without eFluor450-labeled Treg (ratio 1:1) and stimulated with 0.5 µg/ml anti-CD3 mAb in presence or absence of IL-6. Proliferation was assessed on day 3 after excluding 7-AAD^+^ dead cells. Analysis was performed on LSRII (BD Bioscience) and evaluated using DIVA software (BD Bioscience). For some experiments transwell chambers were used to separate MS-Teff (in upper chamber) from Teff and Treg of HC (lower chamber). Cells were stimulated with 0.5 µg/ml anti-CD3 mAb and in case of isolated Teff with T cell-depleted, irradiated PBMC in presence or absence of 30 ng/ml anti-IL-6R mAb Tocilizumab. T cells in upper chamber were stimulated with 0.5 µg/ml anti-CD3 mAb and T cell-depleted and irradiated PBMC in presence or absence of 30 ng/ml Tocilizumab. For some experiments B cell-depleted PBMC were used as Teff, cultured in presence or absence of different Treg ratios and stimulated with 0.5 µg/ml anti-CD3 mAb. Teff proliferation was determined on day three of cultures by addition of 37 kBq/well ^3^H-Tdr for additional 16 h.

### RT-PCR and qRT-PCR

RNA was extracted from 2x10^6^ cells using Rneasy Kit (Qiagen) according to manufacturer's instructions. cDNA was generated by reverse transcription with Sensiscript RT Kit (Qiagen) and amplified by PCR with human IL-6 primer pairs (forward 5′-TTCAATGAGGAGACTTGCCTG-3′, reverse 5′-ACAACAACAATCTGAGGTGCC-3′). Thermocycling parameters began with 94 °C for 1 min. 30 sec. followed by 28-30 cycles: 94 °C 30 sec., 54 °C 45 sec., 72 °C 45 sec. and 7 min. at 72 °C. PCR products were separated on 2 % agarose gels. EF1-α served as control (forward: 5'-gat tac agg gac atc tca ggc tg-3', reverse: 5'-tat ctc ttc tgg ctg tag ggt gg-3'). For some experiments mRNA was analyzed by quantitative RT-PCR (qRT-PCR, 7300 Real Time PCR System (Applied Biosystem) and the QuantiFAST PCR Kit (Qiagen)). Relative IL-2 and IL-6 mRNA (both QuantiTect Primer, Qiagen) expression levels were normalized to EF1-α.

### Statistical analysis

Results represent means plus/minus SEM. Statistical significance of coculture assays was determined using unpaired Student’s t test relative to HC. P-values of less than 0.05 were considered significant and indicated in the corresponding figures (*: p <0.05; **: p <0.01; ***: p <0.001). For some experiments statistical significance was determined by Mann-Whitney-Test. P-values of less than 0.05 were considered significant and indicated in the corresponding figures (*: p <0.05; **: p <0.01; ***: p <0.001). 

## Results

### In MS patients Teff are not efficiently controlled by Treg

In autoimmune patients autoreactive Teff are not efficiently controlled by Treg. The origins of excessive Teff function are controversially discussed in different diseases and ascribed either to impaired Treg function [24] or preactivated Teff [20,25].

**Figure 1:**
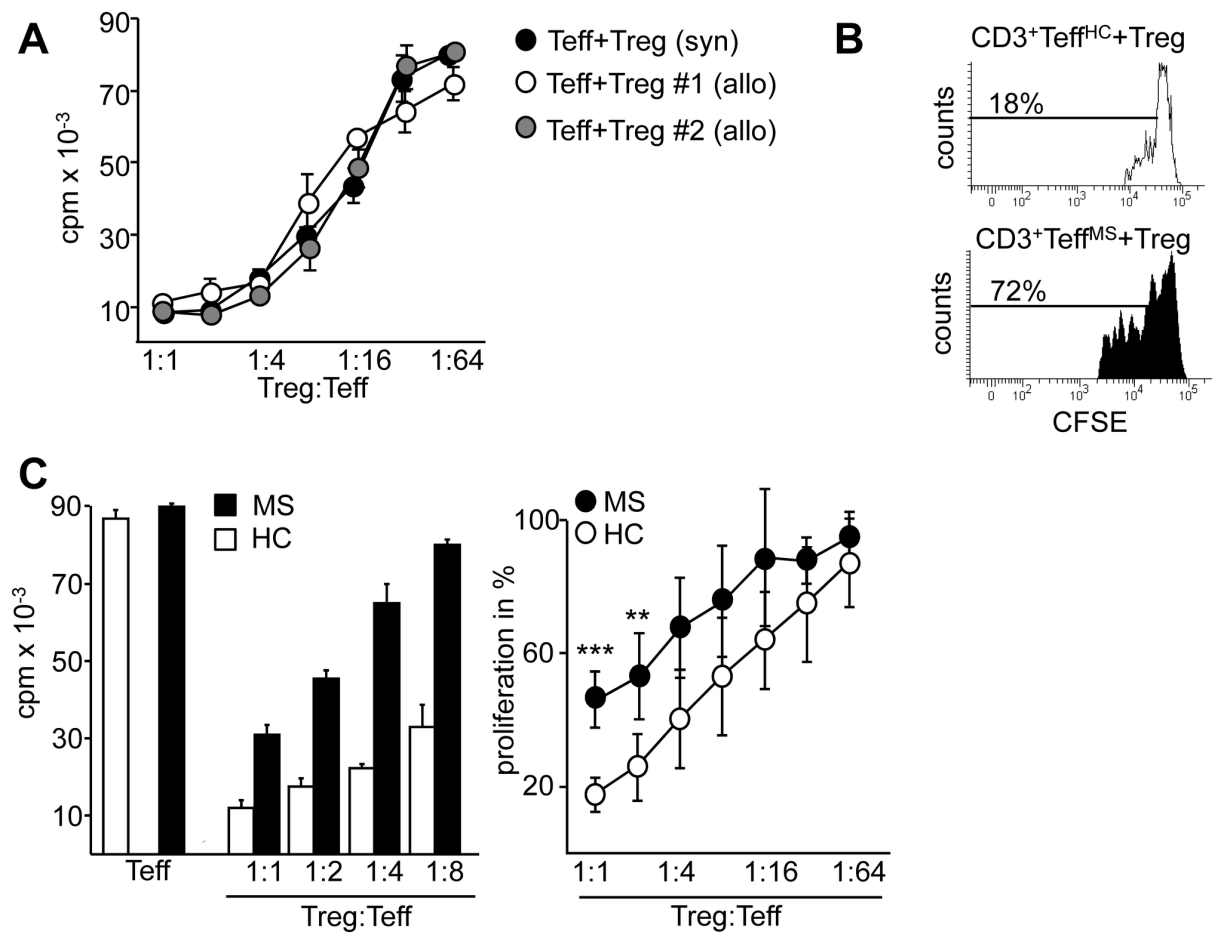
Reduced Treg sensitivity of T effector cells in MS patients is independent of disease course. (A) We defined Teff as Treg-depleted PBMC stimulated with anti-CD3 mAb. Teff from HC were cocultured with syngeneic (black) or allogeneic Treg (white and grey) in different ratios and stimulated with anti-CD3 mAb. Teff proliferation was determined by ^3^H-Tdr incorporation on day three and displayed as mean ± SEM of triplicate measurements. One representative experiment of n=3 is shown. (B) CFSE-labeled Teff from RRMS patients with active disease (black) or HC (white) were cocultured with Treg and stimulated with anti-CD3 mAb. Teff proliferation of CD3^+^ T cells was determined on day three by flow cytometry. One representative result of six independent experiments is shown. (C) Teff from RRMS patients in remission (black) or HC (white) were cocultured with Treg and stimulated with anti-CD3 mAb. Teff proliferation was determined as described. Left: bars represent mean ± SEM of triplicates of one representative experiment. Right: curves show percentage of proliferation in presence of different Treg numbers normalized to proliferation of Teff alone as mean ± SEM of n=28, *P*-values relative to Teff of HC** p<0.01***, p<0.001 are shown.

Based on this background we here focused on the mechanisms of deregulated MS-Teff responses to Treg suppression. Accordingly, as MS patient-derived Treg have been suggested to be functionally impaired, for initial suppressor assays functional Treg from healthy controls (HC) were used. Treg-depleted PBMC (PBMC^depl.^) from therapy-naïve MS patients (Table S 1) [26] provided the Teff populations as well as patient-intrinsic antigen-presenting cells and soluble factors. Since Treg-mediated suppression is antigen-nonspecific and donor-independent (Figure 1A) [22,27], we chose a polyclonal activation of PBMC^depl^ using anti-CD3 mAb as an adequate system to analyze Teff function from MS patients independent of patient intrinsic Treg. Hereby we observed that HC-Treg strongly suppressed Teff proliferation of independent HC but were inefficient in suppression of Teff proliferation from RRMS patients with active disease (Figure 1B). Since MS patients with relapse are exposed to multiple proinflammatory cytokines and chemokines that influence regulatory mechanisms we further analyzed responsiveness of Teff to Treg control from therapy-naïve RRMS patients in remission. Compared to CFSE-based proliferation assays, incorporation of ^3^H-Thymidine is significant more sensitive. Using this assay we observed again an insensitivity of Teff from MS patients in remission to Treg control (Figure 1C) suggesting that unresponsiveness of MS-Teff is probably not influenced by disease activity or course.

### Impaired Teff Control by Foxp3^+^ Treg Mediated by IL-6

**Figure 2:**
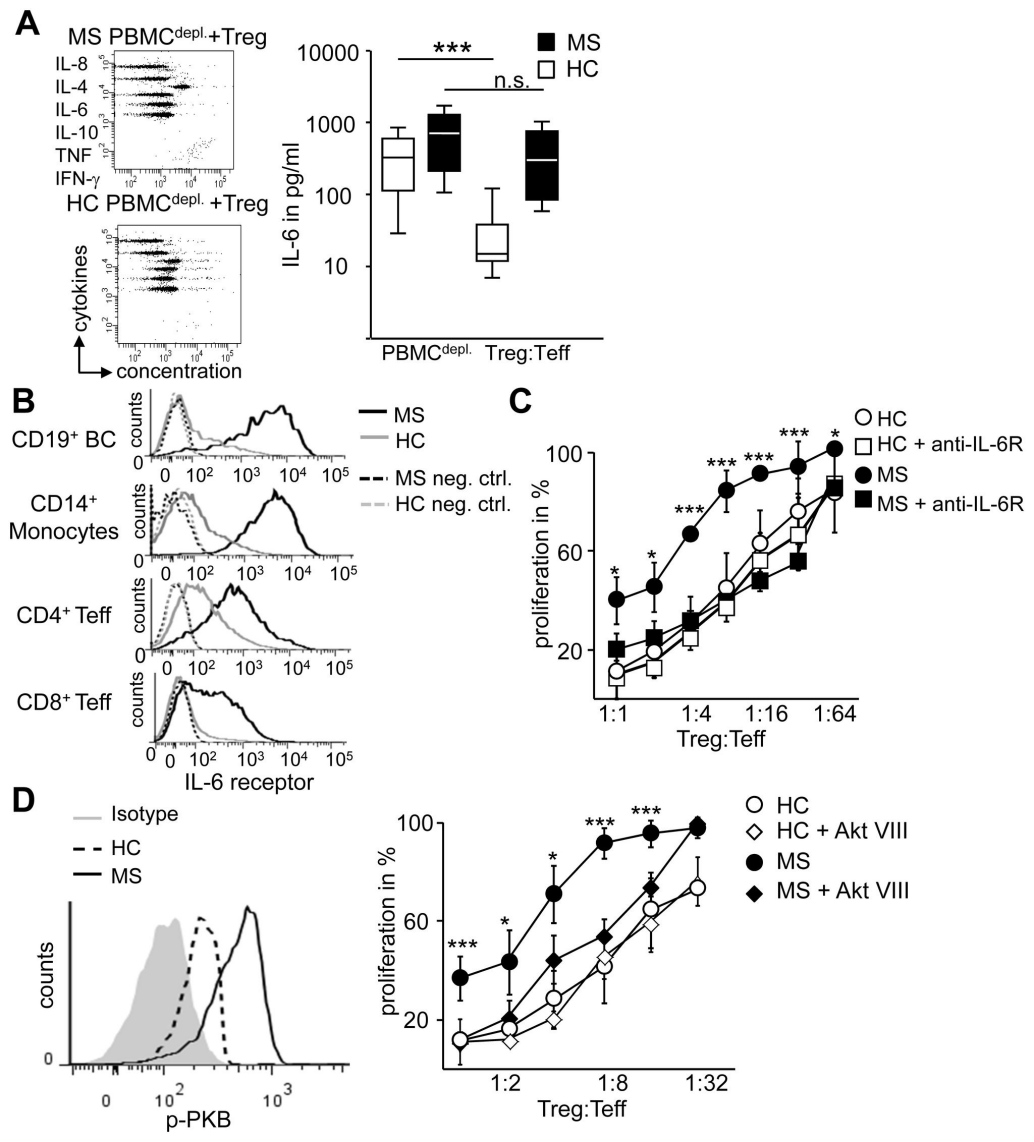
Unresponsiveness of MS-Teff to Treg control is mediated by IL-6. (A) We defined Teff as Treg-depleted PBMC stimulated with anti-CD3 mAb in presence or absence of Treg and determined IL-6 production in supernatants on day three. Boxes represent pooled results of IL-6 secretion of 14 donors from either MS patients (black) or HC (white). Median and interquartile ranges are depicted, statistical significance was determined by Mann-Whitney-test, P-values to Teff ***p<0.001. (B) IL-6R expression within PBMC from HC (grey) or MS (black) was determined by flow cytometry. Negative control as dashed lines, average percentages ± SEM of n=4 different donors are shown. (C) MS-Teff (black) or HC-Teff (white) with Treg were stimulated with anti-CD3 mAb in presence (squares) or absence (circles) of anti-IL6R mAb. Curves show percentage of proliferation in presence of Treg normalized to Teff (n=4), P-values to MS-Teff * p<0.05 ** p<0.01, *** p<0.001. (D) Left: PKB/c-Akt phosphorylation was determined by flow cytometry within CD3^+^ Teff from MS (solid) or HC (dashed). Grey histogram depicts isotypic control of MS. One experiment of n=4 is shown; MS-MFI 366-629; HC-MFI 210-386. Right: MS-Teff (black) with Treg were stimulated with anti-CD3 mAb in presence (diamond) or absence (circle) of Akt-VIII inhibitor. Proliferation of HC (white) served as control. Curves show percentage of proliferation with Treg normalized to Teff (n=4), P-values to MS-Teff * p<0.05, *** p<0.001.

Several proinflammatory cytokines can affect Treg function; we therefore compared cytokine release of activated PBMC^depl^ in presence or absence of HC-Treg. In line with several publications we did not observe significant differences in cytokine release between activated immune cells from MS or HC (Figure S1A). When Treg were added to HC immune cells production of proinflammatory cytokines was strongly decreased. (Figure S1B, Figure 2A links). In general, this was also the case in presence of MS immune cells but with exception of IL-6. This proinflammatory factor was not down regulated in cocultures of MS immune cells and Treg (Figure 2A).

Since Barr et al. recently reported that B cells from RRMS patients secrete elevated amounts of IL-6 [28] we analyzed IL-6 production in more detail. Polyclonal activation of MS immune cells showed only slightly enhanced IL-6 production by CD19^+^ B cells and CD8^+^ T cells (Figure S1C), but without statistical significance compared to HC. More important, IL-6 was considerably down regulated only in suppressor cocultures of HC but not in presence of MS-Teff (Figure 2A) suggesting IL-6 as a potential mediator of failed Treg-mediated suppression. In accordance with this finding we observed a significant higher expression of IL-6R on B cells, monocytes, CD4^+^ and CD8^+^ T cells from MS patients compared to HC (Figure 2B). Consequently, we blocked IL-6 signaling in suppressor cocultures using the clinically approved anti-IL-6R antibody Tocilizumab. Blockade of IL-6R by Tocilizumab had no influence on proliferation of Teff from HC or MS in single cultures. However, in cocultures of MS-Teff with HC-Treg IL-6R blockade rebuild Treg-mediated suppression of MS-Teff whereas suppression of HC-Teff was not influenced (Figure 2C). This demonstrated a link between IL-6 and resistance to Treg function in MS-Teff.

Further analysis of the IL-6 pathway by flow cytometry revealed enhanced phosphorylation of PKB/c-Akt in Teff of MS patients compared to HC (Figure 2D, left). To investigate the impact of PKB/c-Akt phosphorylation on MS-Teff function, a specific PKB/c-Akt inhibitor was added to cocultures that dose-dependently decreased PKB/c-Akt phosphorylation. The inhibitor did not affect proliferative capacity of Teff in response to T cell receptor-mediated stimulation. However, inhibition of PKB/c-Akt activation restored the sensitivity of MS-Teff to Treg-mediated suppression (Figure 2D, right) almost to the level of HC showing that PKB/c-Akt phosphorylation is crucially involved in Treg resistance of MS-Teff.

### IL-6 overcomes Teff suppression without affecting function of Treg

**Figure 3:**
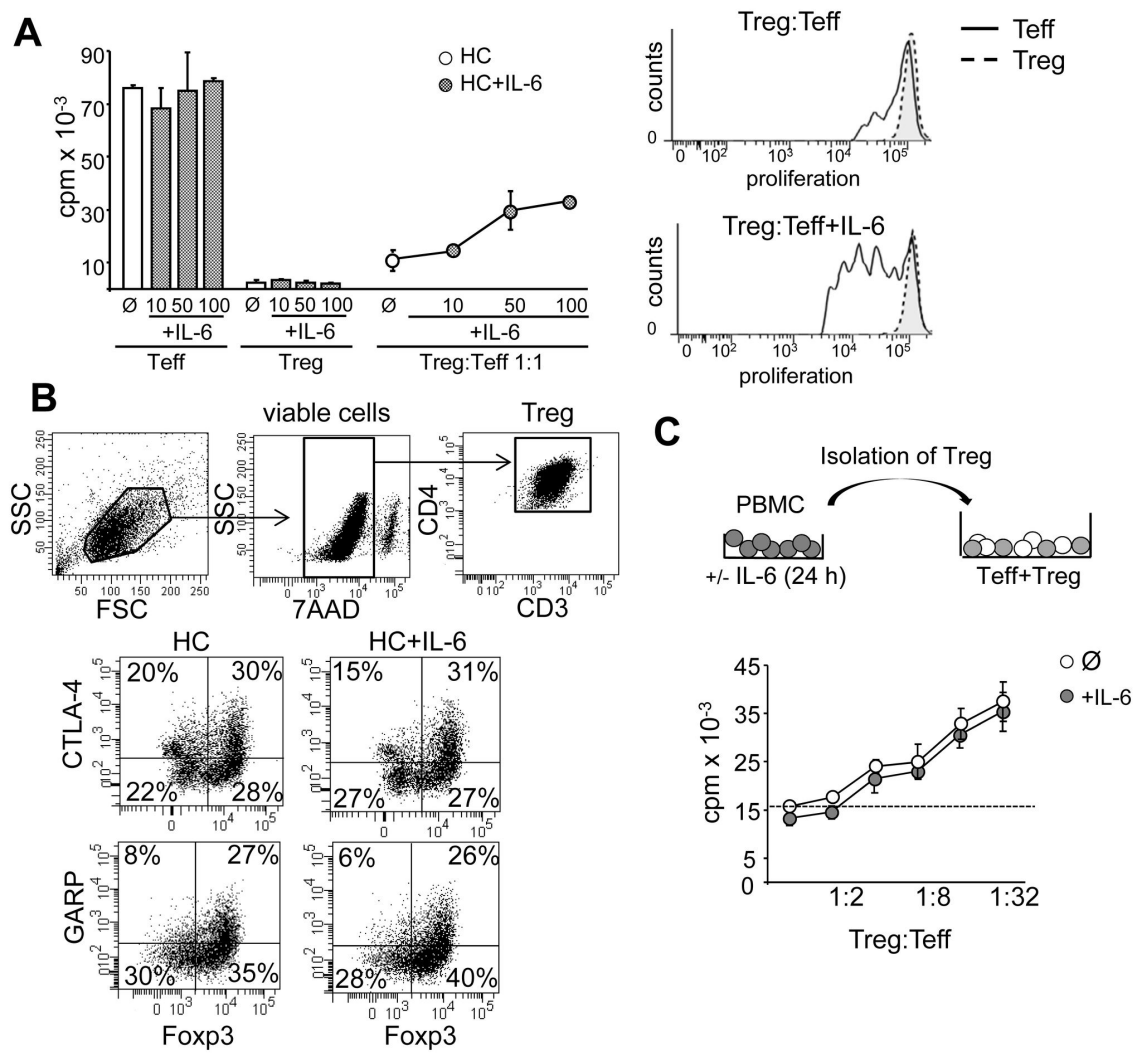
IL-6 renders Teff of HC insensitive to suppressive activity of Treg without affecting their anergic state. (A) We defined Teff as Treg-depleted PBMC stimulated with anti-CD3 mAb. Left: Teff and Treg were cultured alone or in coculture and supplemented with increasing amounts of IL-6 (grey) or left untreated (white). Proliferation was determined by ^3^H-Tdr incorporation on day three and displayed as mean ± SEM of triplicate measurements. One of four independent experiments is shown. Right: CFSE-labeled Teff were stimulated with anti-CD3 mAb in presence or absence of IL-6 and cocultured with eFluor450-labeled Treg. Proliferation of T cell subsets was determined on day three by flow cytometry. One representative result of four independent experiments is shown. (B) Isolated CD4^+^CD25^+^Foxp3^+^ Treg from HC were stimulated with plate-bound anti-CD3 and soluble anti-CD28 mAb in presence or absence of IL-6. After 48 h viable cells were stained for CD3, CD4 and surface expression of CTLA-4 or GARP and nuclear expression of Foxp3 was determined by flow cytometry. (C) PBMC from HC were precultured for 24 h with (grey) or without (white) IL-6, washed extensively, followed by isolation of CD25^+^ Treg. Isolated Treg were cocultured with Teff and stimulated with anti-CD3 mAb. Proliferation was determined by ^3^H-Tdr incorporation on day three and displayed as mean ± SEM of triplicate measurements. One of four independent experiments is shown.

To investigate the influence of IL-6 on function of Treg and Teff separately, we added increasing amounts of IL-6 to anti-CD3 mAb stimulated cultures of Teff and Treg both from HC. Supplementation with IL-6 dose-dependently overcame Treg-mediated suppression in coculture without affecting proliferative capacities of Treg or Teff in single cultures, demonstrating that the anergic state of Treg was not affected by IL-6 (Figure 3A).

Functional Treg activation is associated with up regulation of specific surface molecules such as glycoprotein-A repetitions predominant (GARP) and cytotoxic T lymphocyte antigen-4 (CTLA-4). To analyze whether this phenotype is affected by IL-6 we analyzed Treg activation state and function in presence or absence of IL-6. We found that neither expression of the Treg-specific transcription factor Foxp3 nor expression of activation markers such as GARP or CTLA-4 were modulated by IL-6 (Figure 3B). Additionally, preculture of Treg with high amounts of IL-6 did not alter their suppressive properties (Figure 3C) demonstrating that IL-6 did not affect Treg function in general.

### Accelerated IL-6 synthesis in Teff correlates with their Treg resistance

**Figure 4:**
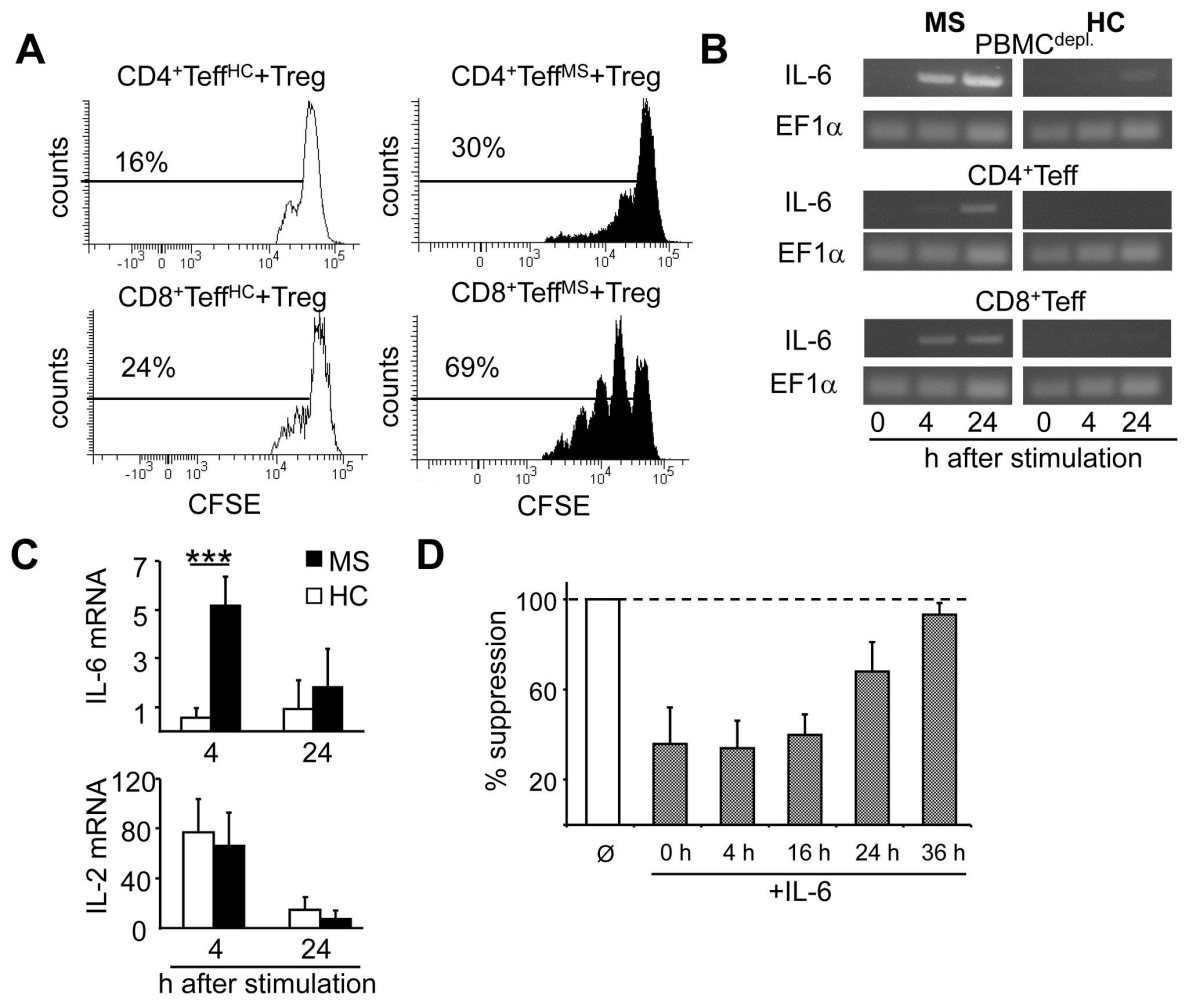
Accelerated IL-6 synthesis is responsible for direct and bystander Treg resistance. (A) Isolated CFSE-labeled Teff of either HC (white) or MS patients (black) were cocultured with HC-Treg of an independent donor (ratio of 1:1) and stimulated with anti-CD3 mAb and T cell-depleted, irradiated PBMC. Proliferation of CD4^+^ (upper panel) or CD8^+^ Teff (lower panel) was determined within CD3^+^ T cells on day three. Percentage of proliferating cells is indicated; one experiment (n=3) is shown. (B) Treg-depleted PBMC, CD4^+^ or CD8^+^ Teff from HC or MS were left unstimulated or were activated with plate-bound anti-CD3 and anti-CD28 mAb. Expression levels of IL-6 mRNA were detected by RT-PCR. EF1-α used as housekeeping gene. One experiment (n=9) is shown. (C) Treg-depleted PBMC from HC or MS were stimulated with plate-bound anti-CD3 mAb and IL-6 or IL-2 mRNA levels were detected by qRT-PCR. EF1-α served as housekeeping gene. Bars represent mean of eight independent experiments; statistical analysis was determined by Mann-Whitney-test, measurements in duplets ***p<0.001. (D) We defined Teff as Treg-depleted PBMC stimulated with anti-CD3 mAb. Teff were cocultured with Treg and stimulated with anti-CD3 mAb in absence (white) or time-delayed addition (grey) of IL-6. Proliferation was determined by ^3^H-Tdr incorporation on day three and displayed as mean suppression ± SEM of triplicate measurements. One experiments of n=4 is shown.

Presence of IL-6 during T cell activation impacts functional interaction of Teff and Treg. Up to now suppressor assays were performed using Treg-depleted PBMC as Teff. Hence, from here we used defined T cell populations isolated from MS patients or HC to investigate Teff function independent of patient-derived APC. Both, isolated CD4^+^ and CD8^+^ Teff from MS patients showed reduced responsiveness to Treg-mediated suppression compared to HC (Figure 4A). Treg resistance in MS is linked to IL-6 and restored by IL-6 blockade. Since both, activated immune cells from HC and MS, produced comparable amounts of IL-6 (Figure 2A and Figure S1A); the abundance of this cytokine in cocultures did not explain Treg unresponsiveness of MS-Teff. However, when we analyzed the kinetics of IL-6 production, we found that in Teff of HC IL-6 mRNA was barely detectable within first 24 h after activation. In contrast, MS-Teff showed an accelerated production of IL-6 mRNA detectable already 4 h after activation (Figure 4B). Interestingly, IL-6 production was most pronounced in CD8^+^ Teff and correlated with their prominent Treg insensitivity compared to CD4^+^ Teff. These results suggested that a disturbed IL-6 kinetics instead of elevated IL-6 synthesis is responsible for Treg resistance of MS-Teff. IL-6 did not modulate IL-2 mRNA synthesis, demonstrating that loss of Treg sensitivity was not provoked through IL-6-induced IL-2 synthesis in MS-Teff (Figure 4C). In order to test our hypothesis, IL-6 was added at different time points to suppressor cultures of HC. Again, IL-6 present from culture start prevented Teff suppression (Figure 4D). 24 h later, IL-6 was not further able to interfere with Treg function (Figure 4D) showing that presence of IL-6 at early processes of T cell activation is essential to induce Treg resistance.

### Early IL-6 by MS-Teff conveyed Treg resistance to HC-Teff

It is well established that cytokines like TGF-β are capable to convey suppressor activity to conventional CD4^+^ T cells in a process described as infectious tolerance [3]. We proposed that IL-6 can play an opposing role in immune regulation by spreading resistance to Treg-mediated control among T cells in a process we named “bystander resistance”.

**Figure 5:**
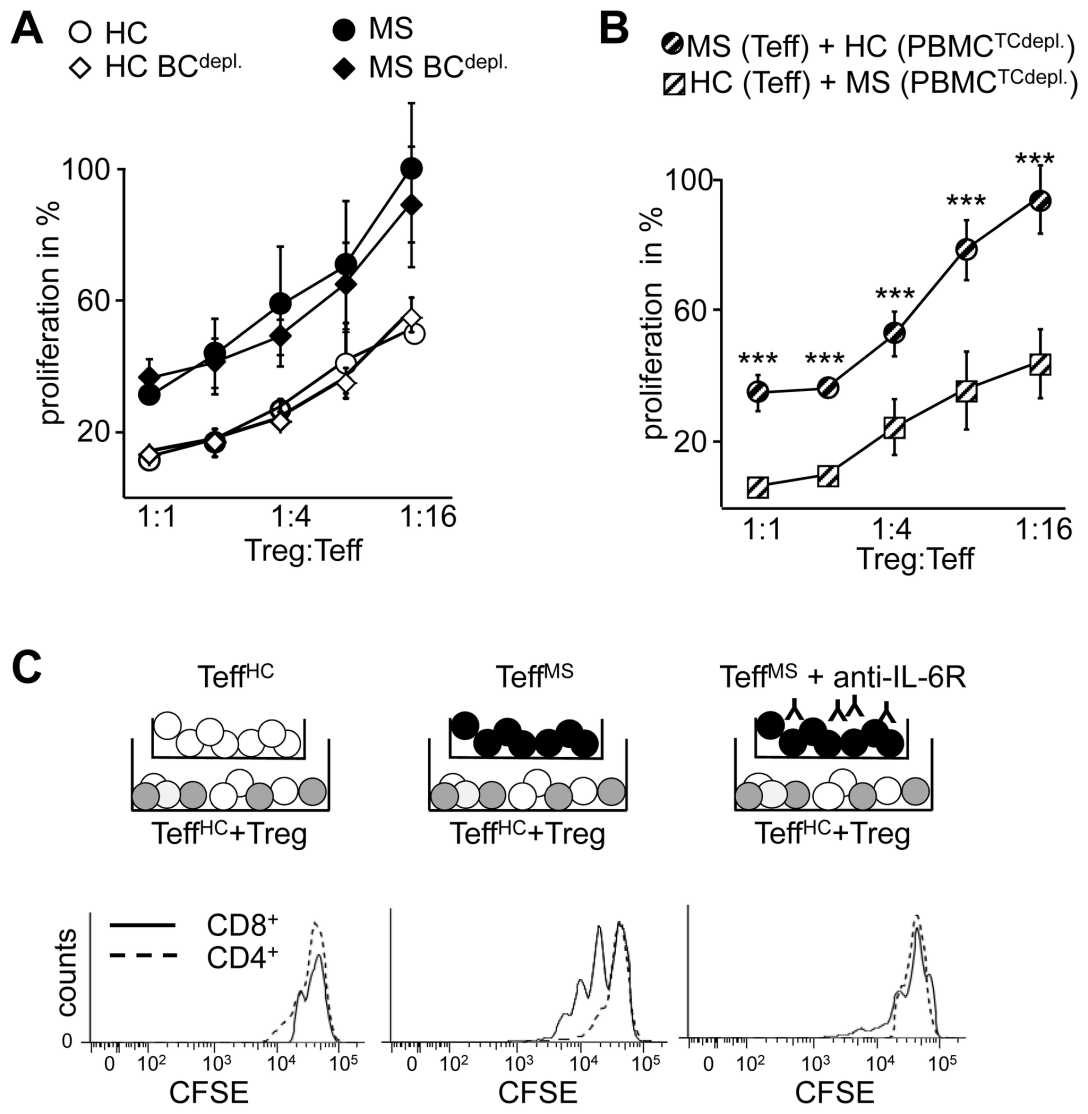
Early IL-6 production by Teff from MS patients induces bystander T cell resistance in surrounding Teff. (A) B cell-depleted PBMC were cocultured with Treg in different ratios and stimulated with anti-CD3 mAb. Teff proliferation was determined by ^3^H-Tdr incorporation on day three and displayed as percentage of proliferation normalized Teff alone as mean ± SEM of four different experiments. (B) Replacement of APC: isolated Teff were cocultured with Treg and stimulated with anti-CD3 mAb in presence of T cell-depleted irradiated PBMC: isolated MS-Teff + Treg + HC PBMC^TCdepl^ (black and white striped circles); isolated HC-Teff + Treg + *MS* PBMC^TCdepl^ (black and white striped quadrats). Proliferation was determined by ^3^H-Tdr incorporation on day three and displayed as percentage of proliferation normalized Teff alone as mean ± SEM of four different experiments, *P*-values relative to HC-Teff*** p<0.001 are shown. (C) Isolated CFSE-labeled Teff of HC (white) were cocultured in lower chamber of transwell experiments with Treg (grey, ratio 1:1) and stimulated with anti-CD3 mAb and T cell-depleted, irradiated PBMC of an independent donor. Isolated Teff from HC (white) or MS patients (black) were added into upper chamber of transwell experiments. In one approach blocking mAb against IL-6R (Tocilizumab) was added into cocultures. Proliferation of CD4^+^ (dashed line) or CD8^+^ Teff (solid line) in lower chamber was determined on day 3. One of three independent experiments is shown.

As we observed that IL-6 is also produced by B cells (Figure S1C) we investigated whether IL-6-producing B cells are accounted to mediate Treg unresponsiveness of MS-Teff. Experiments with B cell-depleted PBMC revealed that B cells were not essential for Treg resistance (Figure 5A). Isolated Teff from MS patients cocultured with Treg and T cell-depleted PBMC of HC were also resistant to Treg-mediated suppression (Figure 5B). 

To test whether IL-6 mediate bystander resistance, we performed transwell experiments. Transwell prevents direct cell contact but allow transition of soluble factors. Since we identified MS-Teff as the responsible source for early IL-6 production within PBMC that mediate Treg resistance, we placed isolated MS-Teff in the upper chamber of transwell. Cocultures of HC-Treg and isolated CFSE-labeled HC-Teff were transferred to lower transwell chambers. Indeed, presence of MS-Teff in the upper chamber was sufficient to overcome HC-Treg-mediated suppression of HC-Teff located in lower chamber (Figure 5C), whereas activation of HC-Teff in upper chamber had no effect. Treg function was restored by blocking antibodies against IL-6R (Figure 5C, right). As shown in Figure 4A CD4^+^ and CD8^+^ Teff were insensitive against Treg-mediated suppression. Surprisingly, in case of bystander resistance through IL-6, CD8^+^ Teff seemed to be more sensitive to IL-6 modulation than CD4^+^ Teff (Figure 5C, middle). Thus, bystander resistance is indeed cell contact independent. IL-6 produced by MS-Teff is responsible for their Treg resistance and further conveys Treg unresponsiveness to surrounding T cells.

### Positive IL-6 feedback loop accelerates IL-6R expression, IL-6 production and mediates bystander resistance

**Figure 6:**
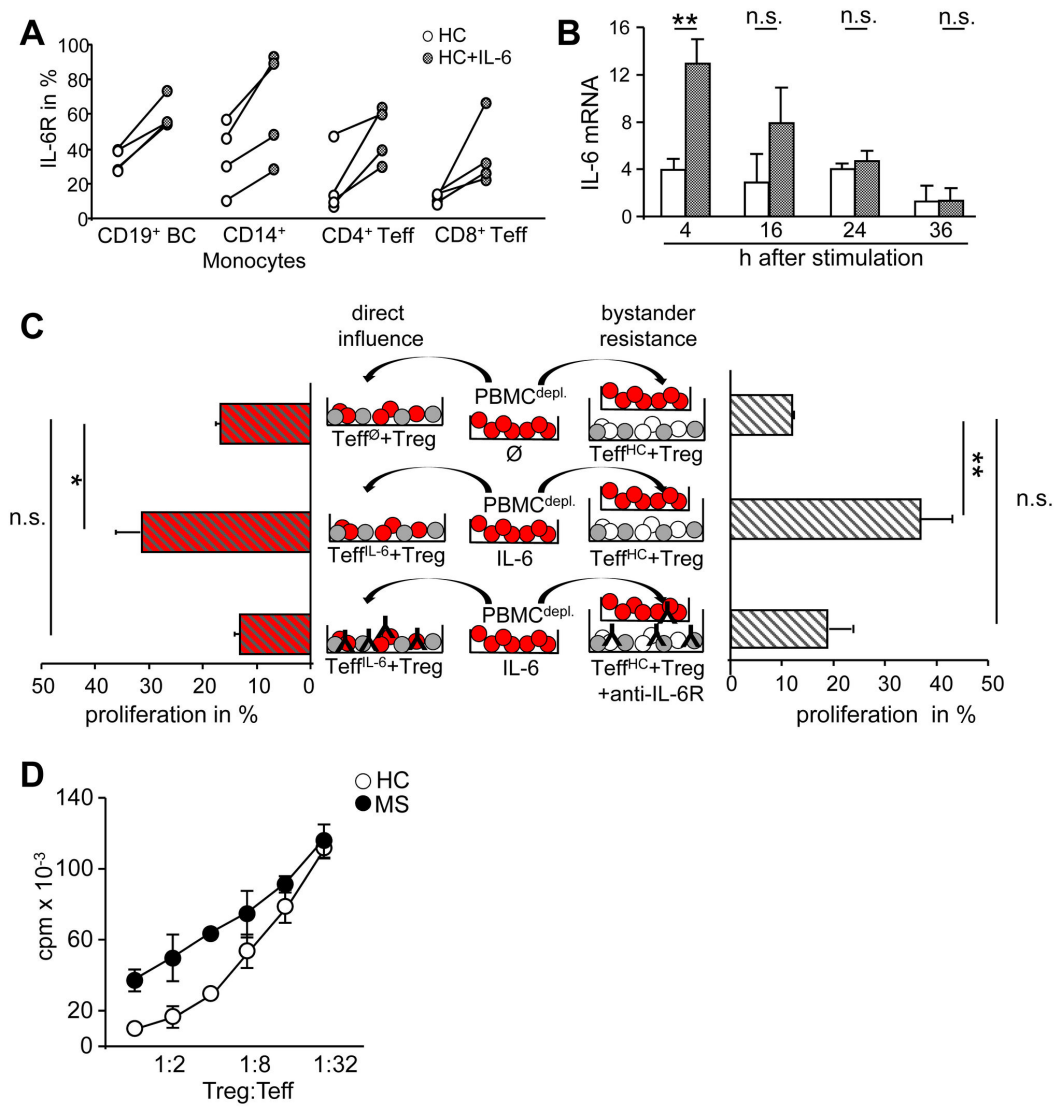
In a positive feedback loop IL-6 induces IL-6R upregulation and accelerated IL-6 production. (A) IL-6R expression after 24 h of culture with (grey) or without IL-6 (white) was analyzed by flow cytometry. Each point represents percentage of IL-6R^+^ cells within CD19^+^, CD14^+^, CD4^+^ or CD8^+^ cells from independent HC (n=4), differences were n.s.. (B) PBMC^depl^. from HC were cultured for 24 h with (grey) or without (white) IL-6, washed extensively, stimulated with plate-bound anti-CD3 mAb and used for qRT-PCR. EF1-α served as housekeeping gene. Statistical analysis was determined by Mann-Whitney-test, *P*-values relative to Teff without IL-6 preincubation ** p<0.01. Bars represent mean of four independent experiments. (C) Left, IL-6 precultured HC PBMC^depl^ (red) were washed, cocultured with HC-Treg (grey) and stimulated with anti-CD3 mAb. Right, Teff (white) were cocultured in the lower chamber with Treg both from HC. IL-6 pre-cultured PBMC^depl^. (red) were washed and placed in upper chamber. Culture was stimulated with anti-CD3 mAb in presence or absence of anti-IL-6R mAb. Proliferation in lower chamber was determined by ^3^H-Tdr incorporation and displayed as percentage of proliferation normalized to Teff alone as mean ± SEM (n=4), *P*-values to cocultures ** p<0.01. (D) Teff from HC or MS were cultured for 24 h in absence of IL-6, cocultured afterwards with Treg and stimulated with anti-CD3 mAb. Proliferation was determined on day three and displayed as mean ± SEM of (n=3) triplicate measurements.

Summarizing our observations we found an altered kinetics of IL-6 synthesis and strong expression of IL-6R in MS patients that is directly linked to Treg resistance of MS-Teff. IL-6 as a pleiotropic proinflammatory cytokine influences the function of several immune cell subsets. Next we investigated the impact of IL-6 on Teff function from healthy controls by analyzing the kinetics of IL-6 synthesis and IL-6R expression in presence of IL-6. Therefore we precultured Treg-depleted HC PBMC in presence of IL-6 without additional stimulation. This preincubation induced strong IL-6R up regulation (Figure 6A) and transient accelerated IL-6 mRNA synthesis (Figure 6B). Preliminary tests exhibited that IL-6 preincubation for at least 8 h is required to modulate Teff function. Interestingly, these IL-6-precultured Teff were Treg resistant (Figure 6C, left). Blockade of IL-6 signaling restored Treg responsiveness, demonstrating that IL-6 is able to induce Treg resistance in Teff from HC (Figure 6C, right). Further transwell experiments showed that these Treg resistant Teff in upper chamber also abolished Treg-mediated suppression of untreated Teff in lower chamber as observed before in presence of MS-Teff. Again, (Figure 6C, right) blockade of IL-6 signaling by supplementation with anti-IL-6R mAb prevented this bystander resistance.

Since we observed that the modulating effect of IL-6 on HC immune cells was only transient (Figure 6B), we analyzed whether this is also the case for MS-Teff after withdrawal of IL-6. We therefore cultured MS PBMC^depl^. in absence of IL-6 for 24 h and transferred these cells to suppressor cultures with HC-Treg. Sustained Treg resistance of MS-Teff despite 24 h preculture in absence of IL-6 (Figure 6D) suggesting an altered IL-6 susceptibility of MS-Teff compared to healthy donors. Whereas IL-6 modulates Teff function of HC only transiently, Teff of MS patients are affected perseverative. This might be an explanation for excessive immune responses in autoimmune MS patients in correlation with the common function of IL-6 in healthy volunteers.

## Discussion

In this study, we analyzed the mechanisms of IL-6-mediated T effector cell (Teff) unresponsiveness to Treg-mediated suppression in RRMS patients that are in remission. We observed that early secretion of IL-6 by Teff of MS patients promoted protein kinase B (PKB)/c-Akt phosphorylation and thereby render these T cells insensitive to Treg control independent of their disease activity. More important, we found that IL-6 itself enhances the expression of its own receptor (IL-6R) and accelerates IL-6 mRNA and protein production through a positive feedback loop, leading to direct and bystander Treg resistance. These results are conductive to the understanding of IL-6-associated pathology in T cell-mediated autoimmune diseases.

Dysregulation of Teff has been assumed to contribute to several autoimmune diseases [25,29,30] and was ascribed to different mechanisms. In MS, inflammatory activity of autoreactive Teff has mainly been attributed to impaired Treg function [31-33] whereas in EAE (experimental autoimmune encephalomyelitis, the corresponding mouse model of MS) a pivotal role of IL-6 in disease development and maintenance was suggested [16,34]. Recently, B cells and dendritic cells were considered as crucial sources of IL-6 [28,35,36]. This was further supported by an ameliorated disease course after depletion of B cells [28]. Our data demonstrated that early IL-6 induces Treg resistance in Teff. Although both activated immune cells from HC and MS produced comparable amounts of IL-6 the proposed mechanism does not exclude that B cells or dendritic cells are the primary source of IL-6 in EAE and MS [28,35,36]. We further propose that initial IL-6 would induce a positive feedback loop of increased IL-6R expression and accelerated IL-6 secretion that now maintain IL-6 production and serve as a secondary source of IL-6 correlating with Treg unresponsiveness. Since the underlying mechanisms of persistent IL-6 maintenance in autoimmunity remained so far elusive these observations uncover a potential explanation. Our thesis is further supported by observations made in lung and breast cancer patients where secreted IL-6 triggered its own production [37].

The finding that IL-6 is coproduced by Th17 cells is in line with the finding that IL-17A promotes IL-6 secretion [38] and underpins the general synergistic correlation between IL-6 and IL-17 in autoimmune diseases [39]. Besides the importance of IL-6 in EAE and its link to the pathogenesis of various other autoimmune disorders [40], influence of IL-6 on MS is controversially discussed. Our results demonstrate for the first time that an altered IL-6 kinetics instead of elevated IL-6 levels [20] is essential for prolonged Treg resistance of MS-Teff independent of the course of disease. Analyzing the kinetics of IL-6 production, we found that IL-6 mRNA in T cells of HC was almost undetectable within 24 h after activation, whereas in MS immune cells and here especially in Teff, a fast synthesis of IL-6 mRNA was observed early after activation. However, this altered IL-6 kinetics is also transiently induced in T cells of HC demonstrating the positive feedback loop of IL-6. This is of particular interest, as we showed that IL-6 affects Treg sensitivity only when present at early time points of T cell activation. On the other hand, IL-6 has virtually no effect when Treg suppression already ensued. Although IL-6 levels in sera of MS patients are not elevated in agreement with the resting state of peripheral T cells in MS patients [18], our results highlight a direct relevance of altered IL-6 kinetics to dysregulated immune responses in MS. Enhanced IL-6R expression and early IL-6 synthesis might synergize to a state of T cell pre-activation that correlates with insensitivity to Treg-mediated control. Additionally, whereas accelerated IL-6 production in T cells of healthy volunteers was down regulated within 24 h in absence of IL-6, in MS-Teff fast IL-6 synthesis maintained also in absence of IL-6 suggesting a MS-specific T cell dysregulation.

Our thesis that a state of Treg resistance is mediated through accelerated IL-6 production in T cells of MS patients is underlined by observations made in rheumatoid arthritis patients. Here, phosphorylation of PKB/c-Akt has been shown to correlate with resistance of Teff to Treg-mediated suppression, a fact that could also be reproduced using murine models [25,41,42]. Since we observed that blockade of IL-6 signaling and usage of PKB/c-Akt inhibitors restored susceptibility of Teff to Treg function, we suggest that protein kinase B signaling is an essential pathway in IL-6-mediated Treg resistance.

In infectious immune responses IL-6 stimulates the acute-phase reaction to support innate immunity and protect against tissue damage [4]. On the other hand, IL-6 is also controlled by negative regulators like suppressor of cytokine signal (SOCS) [43,44] to prevent sustained immune reactions. Since we observed a perseverative unresponsiveness of Teff to Treg control only in MS patients, we propose that negative regulation of IL-6 signaling is disturbed in this autoimmune disorder. We further demonstrate that IL-6 - once induced - mediates Teff insensitivity to Treg suppression independent of antigen presenting cells or impaired Treg function and affects T cells irrespectively of their antigen specificity. This antigen nonspecific affection of T cell function is in agreement with data showing that impaired regulation of inflammatory cytokines like IL-6 maintains pathologic Teff activation in rheumatoid arthritis independent of T cell antigen specificity [45]. Treg-mediated regulation is strictly dependent on their activation [22,46] and is further critically influenced by the activation state of responder T cells: strongly activated T cells cannot be suppressed by otherwise functional Treg [25,30]. We exhibited that presence of IL-6 in suppressor coculture assays did not affect functional activation of Treg indicating that IL-6 principally acts on Teff site. Consistently, observations made in transgenic mice constitutively expressing IL-6 showed that overproduction of IL-6 did not inhibit development or function of Foxp3^+^ Treg *in vivo* [12].

Treg-oriented therapies like IFN-γ and IFN-β treatments enhance Treg frequency and function in MS patients [47,48] but showed only minor effectiveness. Failure of these initial therapies can be explained by our findings showing an IL-6 induced Treg resistance of pathologic T cells. We therefore propose that a combinatory therapy addressing Treg dysfunction and restoration of Teff sensitivity might be more successful. 

The anti-IL-6R antibody Tocilizumab has yielded good responses in clinical trials for rheumatoid arthritis and Crohn's disease [15,49], but no experiences with Tocilizumab in MS therapy are published. Recently, two case series demonstrated that treatment with anti-IL-6R mAb has positive effects in neuromyelitis optica spectrum disorders in humans, further underlining the relevance of IL-6 in pathogenesis of CNS demyelinating disorders [50].

In summary, our results provide strong evidence that altered IL-6 kinetics especially by Teff of MS patients mediates Treg resistance in these T cells. This accelerated IL-6 production induces a positive feedback loop resulting in IL-6R up regulation as well as direct and bystander Treg resistance demonstrating the relevance of the proinflammatory cytokine IL-6 as a potent modifier of early Treg-T cell communication.

## Supporting Information

Figure S1 **Activated immune cells of HC or MS patients show comparable cytokine production.** (A) We defined Treg-depleted PBMC stimulated with anti-CD3 mAb as Teff. Teff of HC (white) or MS (black) were stimulated with anti-CD3 mAb. On day three after stimulation cytokine release (IL-1 IL-2, IL-4, IL-6, IL-8, IL-10, IL-17A, IFN-γ, and TNF) was determined in supernatants. Pooled results of 10 independent donors from either MS (black) or HC (white) in single cultures (A) and cocultures (B) are shown. In case of IL-6 levels 14 donors were measured. Median and interquartile ranges are depicted, statistical analysis was determined by Mann-Whitney-test, *P*-values relative to HC *p<0.05 are shown. (B) Treg-depleted PBMC were stimulated with anti-CD3 mAb in presence of Treg (ratio 1:1). On day three after stimulation cytokine release was determined in supernatants. P-values relative to HC ***p<0.001 are shown. (C) Treg-depleted PBMC of HC or MS were stimulated with PMA and Ionomycin and analyzed by intracellular flow cytometry. Viable cells were stained for CD3^+^CD4^+^, CD3^+^CD8^+^ or CD19^+^ cells. Left: dot plots show percentage of IL-6-producing CD19^+^ B cells, CD8^+^ or CD4^+^ Teff from HC or MS. Right: distribution of IL-6-producing CD19^+^ B cells, CD8^+^ or CD4^+^ Teff from HC (white) or MS (black) are shown, each dot represents an individual donor (n=9). Statistical analysis was determined by Mann-Whitney-test, differences between MS and HC were not significant.
(TIF)

Table S1 **Clinical characteristics of multiple sclerosis patients.** PBMC were collected in heparinized tubes from 51 patients with a relapsing-remitting course (RRMS, age 21 to 64 years). Three patients from Muenster, five from Heidelberg and 43 from Mainz were included in this study. Six patients with RRMS showed a relapse, remaining patients were in remission. Expanded Disability Status Scale (EDSS) was used to quantify disability (0-6). We did not detect any differences in T cell responses regarding the course of disease. All patients had not received previous treatment six months before time point of analysis and were clinically stable. According to the principles expressed in the Helsinki Declaration and to the ethics committee-approved protocols patients provided written informed consent before participating in this study.
(TIF)
