# Correction: Kinetics of IL-6 Production Defines T Effector Cell Responsiveness to Regulatory T Cells in Multiple Sclerosis

**DOI:** 10.1371/annotation/0e76c09e-75b2-493a-90fd-cda3187a0888

**Published:** 2013-11-19

**Authors:** Bettina Trinschek, Felix Lüssi, Jürgen Haas, Brigitte Wildemann, Frauke Zipp, Heinz Wiendl, Christian Becker, Helmut Jonuleit

The name of the second author was spelled incorrectly. The correct name is: Felix Luessi. The correct Citation is: Trinschek B, Luessi F, Haas J, Wildemann B, Zipp F, et al. (2013) Kinetics of IL-6 Production Defines T Effector Cell Responsiveness to Regulatory T Cells in Multiple Sclerosis. PLoS ONE 8(10): e77634. doi:10.1371/journal.pone.0077634. 

